# Supplementary material for: Identification of key somatic oncogenic mutation based on a confounder-free causal inference model
Source: PLoS Comput Biol. 2022 Sep 22;18(9):e1010529. doi: 10.1371/journal.pcbi.1010529 (PMC9499235; doi:10.1371/journal.pcbi.1010529)
Supplement: S1 Text — Section A. Comparison with other methods for discovering single driver mutation. Section B. Parameters analysis of CEBP. Section C. Selection of core genes in CEBP. Table A in S1 Text. The causal effect of TP53 mutation on DNA replication and EMT of BRCA under different numbers of hidden confounders settings. Table B in S1 Text. The causal effect of TP53 mutation on DNA replication and EMT of LUAD under different numbers of hidden confounders settings. Fig A in S1 Text. Fraction of predicted driver genes presents in CGC which consists 616 cancer-related mutations. Fig B in S1 Text. Heat maps of correlations between genes of DNA replication and EMT in BRCA, LUAD, and LIHC tumor samples, where the color indicates the correlation value and the number on the horizontal and vertical axes is the gene’s index. (DOCX) [file pcbi.1010529.s001.docx]

# SUPPORTING INFORMATION

# S1 TEXT

# Section A

# Comparison with other methods for discovering single driver mutation


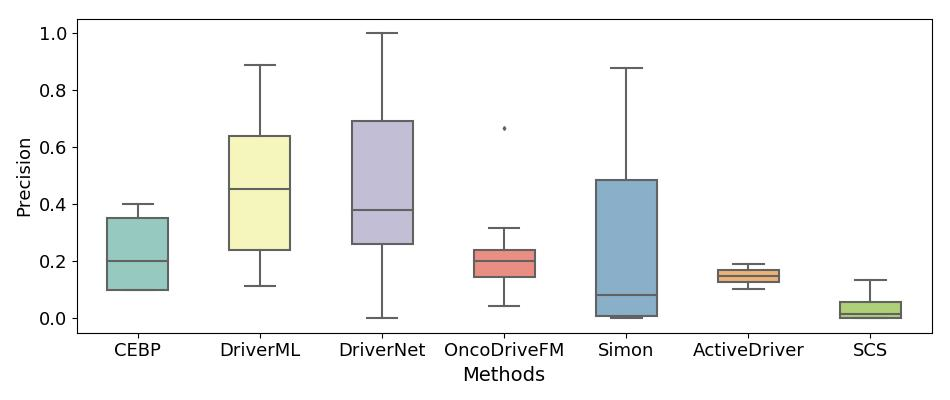


Fig A. Fraction of predicted driver genes presents in CGC which consists 616 cancer-related mutations.

We collect the driver mutation list in Cancer Gene Census (CGC, https://cancer.sanger.ac.uk/census) as the ground truth to validate the key mutation predicted by CEBP for DNA replication. Although driver mutations and key mutations are different concepts and aims, that is the former is for final tumor formation and the latter is for cancer-related important biological processes, we find some identified key mutations are highly consistent with known driver mutations. We only compare predicted results in the DNA replication process, because CGC contains driver mutations that are manually curated or predicted by multiple methods, most of which focus on the cancer cell proliferation process rather than metastasis. Specifically, CEBP evaluates mutations with the top 10 highest ATE values in each cancer whether included in CGC database. The bioinformatics method used for the comparison includes DriverML, DriverNet , OncoDriveFM, Simon, ActiveDriver, and SCS. Predictions of DriverML and SCS are obtained from the Supplementary data of DriverML and predictions of other methods are from DriverDBv2 database (https://pubmed.ncbi.nlm.nih.gov/26635391/). Fig A displays the proportion of predicted mutations that are presented in CGC across 10 cancers. Our approach is comparable to state-of-the-art methods for the discovery of driver mutations and can stable predict driver mutation for each cancer.

# Section B

# Parameters analysis of CEBP

For setting 200 observed confounders in the model, we have two main reasons. First, it is to prevent violations of the positivity assumption in causal inference. Positivity requires that each subgroup of the data with different covariates has a certain probability of getting all the treatment values (for the binary treatment model, the treatment value is 0 and 1). There exists a trade-off between the positivity assumption and the unconfoundedness assumption: although considering more confounders may lead to a higher probability of satisfying the unconfoundedness, it also may lead to a higher probability of violating the positivity. Therefore, we don’t consider all mutations as confounders, but a portion of mutations with high mutation rates as confounders. Secondly, as shown in Table 3, the number of samples and gene mutation frequencies are very different across cancer types, and we attempt to consider these two numbers and make a generic assumption of the number of observed confounders.

Regarding the parameter 20 for hidden confounders in the model, on one hand, it doesn't indicate the absolute number of hidden confounders in the biological system is 20. Actually, we don't exactly know how many and what they are. The hidden confounder is represented by a hidden layer of the VAE model and 20 hidden confounders mean that the hidden layer is 20-dimensional. On the other hand, through conducting a parameter analysis, we find that results are stable and are less affected by parameter values. When setting dimensions of the hidden layer of the model as 10, 20, 30, 40, and 50, respectively, and repeating the experiments 10 times, we calculate the causal effect of TP53 mutation on the DNA replication and EMT in BRCA and LUAD, and the mean and variance of ATE values are shown in Table A and Table B.

Table A. The causal effect of TP53 mutation on DNA replication and EMT of BRCA under different numbers of hidden confounders settings.

| ATE of *TP53* of BRCA on DNA replication | | ATE of *TP53* of BRCA on EMT | |
| --- | --- | --- | --- |
| #Hidden confounder | ATE | #Hidden confounder | ATE |
| 10 | 0.132±0.005 | 10 | 0.044±0.004 |
| 20 | 0.138±0.006 | 20 | 0.048±0.005 |
| 30 | 0.138±0.004 | 30 | 0.042±0.005 |
| 40 | 0.137±0.005 | 40 | 0.047±0.001 |
| 50 | 0.139±0.004 | 50 | 0.043±0.004 |

Table B. The causal effect of TP53 mutation on DNA replication and EMT of LUAD under different numbers of hidden confounders settings.

| ATE of *TP53* of LUAD on DNA replication | | ATE of *TP53* of LUAD on EMT | |
| --- | --- | --- | --- |
| #Hidden confounder | ATE | #Hidden confounder | ATE |
| 10 | 0.123±0.011 | 10 | 0.028±0.005 |
| 20 | 0.135±0.013 | 20 | 0.037±0.006 |
| 30 | 0.125±0.012 | 30 | 0.03±0.006 |
| 40 | 0.143±0.005 | 40 | 0.019±0.008 |
| 50 | 0.129±0.012 | 50 | 0.025±0.008 |

# Section C

# Selection of core genes in CEBP


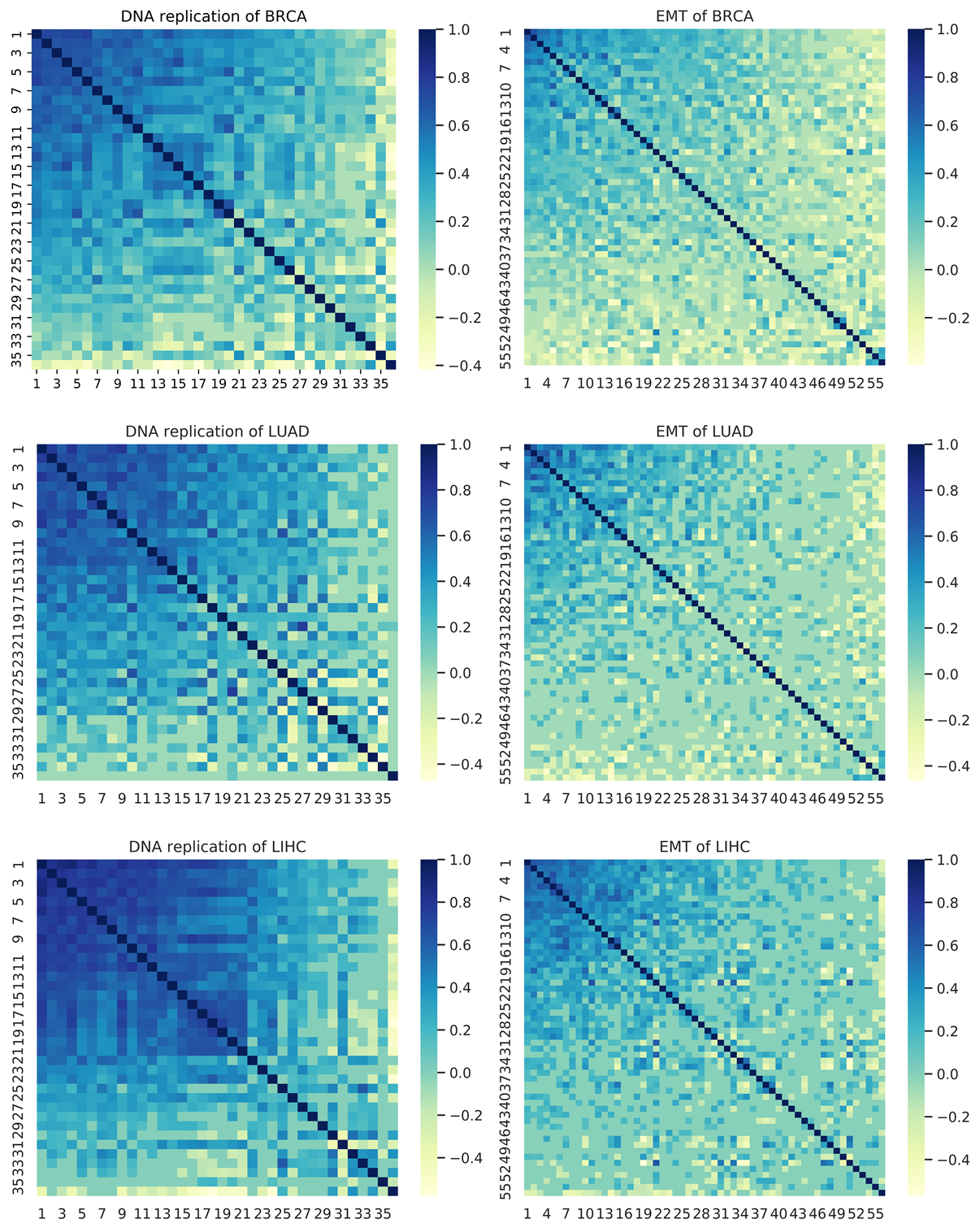


Fig B. Heat maps of correlations between genes of DNA replication and EMT in BRCA, LUAD, and LIHC tumor samples, where the color indicates the correlation value and the number on the horizontal and vertical axes is the gene’s index.

For the active cancer-related biological process, most genes work together and are significantly co-expressed. According to Pearson correlation analysis of the genes in DNA replication and EMT processes of BRCA, LUAD and LIHC tumor samples, as shown in Fig B, more than half of the genes in each biological process are significantly co-expressed. In Fig B, the correlation matrix of each biological process sets p-value =10^-3^ as the cutoff and X-axis and Y-axis represent gene’s index which is ranked by the overall co-expression intensity with other genes. We assume the largest co-expressed module carries the most principal information about the biological process and use it to quantify the relevant activities of the biological process of each sample.
