# Supplementary material for: Identification of key somatic oncogenic mutation based on a confounder-free causal inference model
Source: PLoS Comput Biol. 2022 Sep 22;18(9):e1010529. doi: 10.1371/journal.pcbi.1010529 (PMC9499235; doi:10.1371/journal.pcbi.1010529)
Supplement: Attachment — Submitted filename: Liu_plos_cb_2022_first_review.pdf [file pcbi.1010529.s002.pdf]

The paper by Liu et al. addresses the important problem of identifying causal effects of cancer mutations on downstream processes. Addressing this problem has been the goal of many papers in the past, but utilization of observational data causal inference methods is scarce. This work suggests utilizing a specific graphical model that is learned using variational autoencoders in order to recover and adjust for potential confounders. While we appreciate the goal, and encourage the authors to continue in this direction, we have major concerns that we list below.

The assumed underlying model is not realistic form the addressed biological question

The underlying causal model shown in **Figure 1** assumes that the outcome Y has incoming edges only from the confounders Z or the treatment variable M. Moreover, Z affects some biological variables X that are used to learn the confounding effects by Z. However, when analyzing gene mutation or gene expression data it is extremely unlikely to assume no effects from X to Y as presented in Figure 1. In fact, by analyzing multiple genes as potential treatment effect variables while considering other mutated genes as the potential confounders (as explicitly stated in lines 110-11), the authors support the idea that these X variables may be directly linked to the outcome Y. Thus, the presented method may not correct for confounding effects as implied in the text: see comments after the proof of Theorem 1 in Louizos' original paper about the Y-X independence assumption, and the appendix for why proxy variables may introduce bias. We suggest either exploring how sensitive the method is for these deviations from the underlying model using realistic simulations, or showing why the method should work in additional graphical models that are in line with the presented analysis.

The definition of the outcome (Y) should be better motivated and improved

While we agree with the biological importance of the explored pathways in this paper, we think that the definition of Y should be improved. First, in theory, the proposed method can be run using the expression profile of any target gene g. By exploring all genes the authors can establish if a pathway of interest tends to be enriched among the genes with the strongest causal effects. This will avoid some of the arbitrary decisions made by the current definition of Y that relies on regression analysis of the most correlated gene set within the pathway of interest. We see the current definition as problematic for two reasons: (1) what if the explored pathway P is not highly correlated and can be clustered into two or more groups of co-expressed gene sets? as the presented methods CEBP is introduced as a general method for analyzing new data and not just the presented pathways in the paper, we feel that the assumption that the pathway of interest P must necessarily be driven by a single large group of co-expressed genes should be avoided whenever possible; (2) the identification of the main gene set within the pathway P relies on "Then we choose half the number of the features with higher gcor which can be considered as significant ones", why is this definition correct? This definition means that a set of “significant” genes will appear in any randomly generated data matrix, even if no true signal is present.

The proposed method does not estimate the variance of the causal effects

A major limitation of the proposed method is that it only provides the point estimates but not their variance. Thus, it is difficult to estimate the false discovery rate of a set of selected results. We propose considering bootstrap for estimating the variance of these estimates.

The analysis should cover additional scenarios, datasets, and algorithms

We think that the presented analysis is limited, and fails to establish the benefits of the proposed method. We think that the authors should apply the method to additional pathways or use all genes as we suggest above. Then the authors can show which pathways are likely to be affected by mutations and which do not. Moreover, applying pathway enrichment analysis downstream (e.g., when Y represents single genes) can be used to illustrate how the method can find novel biological insights without the need to select a specific Y in advance.

Another major limitation of the proposed analysis is the lack of references to existing methods. While not directly relying on causal inference of observational data, multiple network biology methods have been proposed in the past for identifying causal connections between gene mutations and gene expression data. Early publications include the ResponseNet algorithm (Yeger-Lotem et al. 2009) and TieDIE (Paull et al. 2013), and additional algorithms have been proposed since. Comparing the output of these established methods with what causal inference methods can provide is of great interest for the community, and we believe will be a natural question for most readers. We also think that these methods should be explained in the introduction, which will help readers in understanding how the problem of identifying driver mutations has been addressed in the past.
