# Supplementary material for: Identification of key somatic oncogenic mutation based on a confounder-free causal inference model
Source: PLoS Comput Biol. 2022 Sep 22;18(9):e1010529. doi: 10.1371/journal.pcbi.1010529 (PMC9499235; doi:10.1371/journal.pcbi.1010529)
Supplement: Attachment — Submitted filename: 2208012Reviewer.docx [file pcbi.1010529.s003.docx]

1. **Reviewer #1:**

1. The authors should give the design philosophy of quantifying the activity of cancer related biological processes. The current description, from line 133 to 137, is not easy to understand.

**Response:** Thanks for your suggestion. As there is no direct biological processes activity reported in the data, so we propose a reliable method to quantify the relative biological processes activity of each sample by performing regression analysis on core genes. We first identify core genes for each biological process according to the significant co-expression pattern. Then, we perform a linear regression analysis of the expression matrix of the core genes on the average expression of core genes in all samples. The coefficient of each sample is regarded as the relative activity. We give a short description from line 133 to 137 in the previous manuscript and the details of the calculation are given in Section 3.2. Following your suggestion, we have updated the manuscript to make the short description clear from line 153 to 159 in the revised version.
2. The authors set 200 observed confounders and 20 hidden confounders in the VAE model. How do these two values be determined?

**Response:** Thanks for this question. For setting 200 observed confounders in the model, we have two main reasons. First, it is to prevent violations of the positivity assumption in causal inference. Positivity requires that each subgroup of the data with different covariates has a certain probability of getting all the treatment values (for the binary treatment model, the treatment value is 0 and 1). There exists a trade-off between the positivity assumption and the unconfoundedness assumption: although considering more confounders may lead to a higher probability of satisfying the unconfoundedness, it also may lead to a higher probability of violating the positivity. Therefore, we don’t consider all mutations as confounders, but a portion of mutations with high mutation rates as confounders. We explain this in line 158-160 of the original manuscript and in line 336-338 of the revised version. Secondly, as shown in Table 3, the number of samples and gene mutation frequencies are very different across cancer types, and we attempt to consider these two numbers and make a generic assumption of the number of observed confounders.

Regarding the parameter 20 for hidden confounders in the model, on one hand, it doesn’t indicate the absolute number of hidden confounders in the biological system is 20. Actually, we don’t exactly know how many and what they are. The hidden confounder is represented by a hidden layer of the VAE model and 20 hidden confounders mean that the hidden layer is 20-dimensional. On the other hand, through conducting a parameter analysis, we find that results are stable and are less affected by parameter values. When setting dimensions of the hidden layer of the model as 10, 20, 30, 40, and 50, respectively, and repeating the experiments 10 times, we calculate the causal effect of *TP53* mutation on the DNA replication and EMT in BRCA and LUAD, and the mean and variance of ATE values are shown below:

| ATE of *TP53* of BRCA on DNA replication | | ATE of *TP53* of BRCA on EMT | |
| --- | --- | --- | --- |
| #Hidden confounder | ATE | #Hidden confounder | ATE |
| 10 | 0.132±0.005 | 10 | 0.044±0.004 |
| 20 | 0.138±0.006 | 20 | 0.048±0.005 |
| 30 | 0.138±0.004 | 30 | 0.042±0.005 |
| 40 | 0.137±0.005 | 40 | 0.047±0.001 |
| 50 | 0.139±0.004 | 50 | 0.043±0.004 |

| ATE of *TP53* of LUAD on DNA replication | | ATE of *TP53* of LUAD on EMT | |
| --- | --- | --- | --- |
| #Hidden confounder | ATE | #Hidden confounder | ATE |
| 10 | 0.123±0.011 | 10 | 0.028±0.005 |
| 20 | 0.135±0.013 | 20 | 0.037±0.006 |
| 30 | 0.125±0.012 | 30 | 0.03±0.006 |
| 40 | 0.143±0.005 | 40 | 0.019±0.008 |
| 50 | 0.129±0.012 | 50 | 0.025±0.008 |

We have added the illustration of these two parameter in line 338 to 340 of the revised manuscript and in Section 2 of S1 text.

3. Regarding the effectiveness of CEBP, it is suggested to compare it with relevant gold standard, and verify the validity of conclusions through wet lab experiments or other credible computational experiments.
**Response:** Thanks for your suggestion. We collect the driver mutation list from Cancer Gene Census (CGC, https://cancer.sanger.ac.uk/census) as ground truth to validate the key mutation predicted by CEBP for DNA replication. Although driver mutations and key mutations are different concepts and aims, that is the former is for final tumor formation and the latter is for cancer-related important biological processes, we find some identified key mutations are highly consistent with known driver mutations and results are shown in Fig. S1 of S1 text.

4. The authors should clarify what the meanings of “measurement of the biological process” in line 129, and “the biological process estimation” in line 132 are. In Section 2.3, the authors should clarify what the meanings of “ a value less than 0 says the treatment can only hurt outcomes and cannot help them” and “the mutation with such ATE is detrimental to biological processes” are.

**Response:** Thanks for this suggestion. The “measurement of the biological process” in line 129 and “the biological process estimation” in line 132 refer to the proposed method for estimating the cancer-related biological process activity of each sample. We have updated this paragraph and given the details of this method in section 3.2 in the revised version. The “ a value less than 0 says the treatment can only hurt outcomes and cannot help them” is intended to illustrate that we consider a mutation with ATE negative value means it doesn’t affect the cancer-related biological process.

5. The source code of CEBP should be provided.

**Response:** Thanks for this suggestion. We have uploaded the code at [https://github.com/awa121/CauMu.](https://github.com/awa121.)

6. Please give More description to Figure 2.

**Response:** Thanks for this suggestion. We have added a more detailed description for Figure 2 in the revised manuscript.

7. Reference 31 is not correct.

**Response:** Thanks for spotting this! We have checked all references and updated the manuscript.

8. The writing should be greatly improved. Some typos should be corrected, such as “biological procession”, “Fig. 3 & & 4 ”, “A a value of ATE equal to 0 ”, etc.

**Response:** Thank you for pointing these out. We have checked the whole article in the revised version. We really appreciate your efforts in reviewing this manuscript.

2. **Reviewer #2:**

1. There are no data and codes on the GitHub page.

**Response:** Thanks for this suggestion. We have uploaded the code and data at [https://github.com/awa121/CauMu.](https://github.com/awa121.)

2. According to the descriptions in section 2.1, X is a 0-1 matrix, M is a 0-1 vector, and is dataset D only used to calculate Y?

**Response:** Thank you for this question. We consider that the confounding is mainly caused by passenger mutations and external factors. X is a 0-1 matrix composed of the somatic mutation data, representing the observed confounders of the causal model. On the other hand, Since some external factors, such as microenvironment stresses, are not measurable but able to lead to changes at the genome level, X is also used as a proxy for the unobserved hidden confounders. M is a 0-1 vector indicating whether the gene mutates or not of samples. Y is the continuous variable presenting the activity of a cancer-related biological process and is calculated from the gene expression dataset D. Compared with somatic mutation data, gene expression is the most fundamental level at which the genotype gives rise to the phenotype and directly reflects the state of the cell. Therefore we use D to indicate the activity of DNA replication and EMT.

3. Equation 1 should be expanded to explain how those expectations are calculated.

**Response:** Thanks for your suggestion. We explain the biological meaning of ATE values and add the description of how we calculate expectations of Equation 1 in line 138-145 in the revised version. The calculation details are in Section 3.3.

4. In section 2.3, the number of observed and hidden confounders is set up to 200 and 20, respectively. Additional explanations and experiments are required to explain the influence of parameters on the performance.

**Response:** Thanks for this question. For setting 200 observed confounders in the model, we have two main reasons. First, it is to prevent violations of the positivity assumption in causal inference. Positivity requires that each subgroup of the data with different covariates has a certain probability of getting all the treatment values (for the binary treatment model, the treatment value is 0 and 1). There exists a trade-off between the positivity assumption and the unconfoundedness assumption: although considering more confounders may lead to a higher probability of satisfying the unconfoundedness, it also may lead to a higher probability of violating the positivity. Therefore, we don’t consider all mutations as confounders, but a portion of mutations with high mutation rates as confounders. We explain this in line 158-160 of the original manuscript and in line 336-338 of the revised version. Secondly, as shown in Table 3, the number of samples and gene mutation frequencies are very different across cancer types, and we attempt to consider these two numbers and make a generic assumption of the number of observed confounders.

Regarding the parameter 20 for hidden confounders in the model, on one hand, it doesn’t indicate the absolute number of hidden confounders in the biological system is 20. Actually, we don’t exactly know how many and what they are. The hidden confounder is represented by a hidden layer of the VAE model and 20 hidden confounders mean that the hidden layer is 20-dimensional. On the other hand, through conducting a parameter analysis, we find that results are stable and are less affected by parameter values. When setting dimensions of the hidden layer of the model as 10, 20, 30, 40, and 50, respectively, and repeating the experiments 10 times, we calculate the causal effect of *TP53* mutation on the DNA replication and EMT in BRCA and LUAD, and the mean and variance of ATE values are shown below:

| ATE of *TP53* of BRCA on DNA replication | | ATE of *TP53* of BRCA on EMT | |
| --- | --- | --- | --- |
| #Hidden confounder | ATE | #Hidden confounder | ATE |
| 10 | 0.132±0.005 | 10 | 0.044±0.004 |
| 20 | 0.138±0.006 | 20 | 0.048±0.005 |
| 30 | 0.138±0.004 | 30 | 0.042±0.005 |
| 40 | 0.137±0.005 | 40 | 0.047±0.001 |
| 50 | 0.139±0.004 | 50 | 0.043±0.004 |

| ATE of *TP53* of LUAD on DNA replication | | ATE of *TP53* of LUAD on EMT | |
| --- | --- | --- | --- |
| #Hidden confounder | ATE | #Hidden confounder | ATE |
| 10 | 0.123±0.011 | 10 | 0.028±0.005 |
| 20 | 0.135±0.013 | 20 | 0.037±0.006 |
| 30 | 0.125±0.012 | 30 | 0.03±0.006 |
| 40 | 0.143±0.005 | 40 | 0.019±0.008 |
| 50 | 0.129±0.012 | 50 | 0.025±0.008 |

We have added the illustration of these two parameter in line 338 to 340 of the revised manuscript and in Section 2 of S1 text.

5. In sections 2.2 and 2.3, a value of ATE equal to 0 means there is no difference in the outcome of the control and treatment group, i.e. a value of ATE not equal to 0 means there is a difference in the outcome. Positive means beneficial, and negative means harmful. So why only consider the positive ATE value, not the absolute value as the causal effect of genes? In this way, the result will be very different from figure 3.

**Response:** For a mutation with negative ATE on a biological process, i.e. ATE=E[Y(t=1)-Y(t=0)]<0, it means that the biological process activity with the mutation is lower than that without the mutation, suggesting the mutation doesn’t promote or benefit this biological process. In this paper, we assume key mutations should have positive effects on cancer-related biological processes, that is mutations with positive ATE. Therefore, we don’t consider using absolute value as the causal effect of mutations. Another reason is that the general view in the biological field is that mutations always have positive effects on cancer biological processes such as DNA replication and EMT, and studies of the harmful or side effects of mutations are very limited. So we don’t conduct in-depth research and analysis on such mutations in this paper, but we will study them in future work.

6. Figure 5 only shows the boxplot of TP53, the boxplot of other genes should be included in supplementary materials.

**Response:** Thanks for your suggestion. We have added boxplots of genes with the highest ATE value and the highest mutation frequency in each cancer, as shown in Fig. 5 to 8 in the revised manuscript.

7. In section3.1, the data processing process is described vaguely and should be described more clearly. Make every step in the preprocessing clear. What software was used?

**Response:** Thanks for your suggestion. We have updated Section 3.1 in the revised version. In this section, we have introduced the selection of genes and candidate key mutations, sample filtration, collection of gene sets of cancer-related biological processes, and selection of observed confounders.

8. In equation 2, the meaning of function  should be further explained.

**Response:** In Eq. 2, $\chi_{(s_{i}^{j}<\alpha)}$ is the indicator function. $\alpha$ is a predefined hyperparameter to drop the correlation parameter with low significance, if $s_{i}^{j}<\alpha$, $\chi_{(s_{i}^{j}<\alpha)}$ is 1; if $s_{i}^{j}\geq\alpha$, $\chi_{(s_{i}^{j}<\alpha)}$ is 0. We have updated the definition of $\chi_{(s_{i}^{j}<\alpha)}$ in Eq. 2 in the revised manuscript.

9. In section 3.2, why choose half the number of the features with higher gcor which can be considered as significant ones?

**Response:** For the active cancer-related biological process, most genes work together and are significantly co-expressed. According to Pearson correlation analysis of the genes in DNA replication and EMT processes, as shown in Fig. 1, we find that more than half of the genes in each biological process are significantly co-expressed. We assume the largest co-expressed module carries the most principal information about the biological process and use it to quantify the relevant activities of the biological process of each sample. We have added the illustration of this parameter in line 328 to 332 of the revised manuscript and in Section 3 of S1 text.

Figure 1. Heat maps of correlations between genes of DNA replication and EMT in BRCA, LUAD, and LIHC tumor samples, where the color indicates the correlation value and the number on the horizontal and vertical axes is the gene’s index.

10. In section 3.3, why choose to set a shared-layer neural network to represent g and unshared-layer neural networks to represent f? What are the benefits of doing this?

**Response:** The inference network is a two-stage model. The first stage is a shared-layer neural network to learn a representation of (X, Y); the second stage has a bifurcation for the treatment variable. When estimating the effect of a certain gene, samples with mutation in this gene are trained by the $f_{1}$ neural network, and samples without mutation in this gene are trained by the $f_{0}$ neural network. The shared-layer neural can learn high dimensional non-linear relationships and the unshared-layer neural network of the second stage can keep the influence of the treatment variable in the training process.

11. In equation 4, a detailed explanation of each probability distribution definition should be given.

**Response:** Thanks for your suggestion. We have updated Section 3.3 in the revised version and explain the probability distribution of Equation 5.

12. More details should be added about your model training, such as experimental settings and parameter selection.

**Response:** Thanks for your suggestion. We have added the parameter illustration of the model in Section 3.4 of the revised manuscript and in Section 2 of S1 text.

3. **Reviewer #3:**

**1 The assumed underlying model is not realistic form the addressed biological question**

The underlying causal model shown in Figure 1 assumes that the outcome Y has incoming edges only from the confounders Z or the treatment variable M. Moreover, Z affects some biological variables X that are used to learn the confounding effects by Z. However, when analyzing gene mutation or gene expression data it is extremely unlikely to assume no effects from X to Y as presented in Figure 1 In fact, by analyzing multiple genes as potential treatment effect variables while considering other mutated genes as the potential confounders (as explicitly stated in lines 110-11), the authors support the idea that these X variables may be directly linked to the outcome Y. Thus, the presented method may not correct for confounding effects as implied in the text: see comments after the proof of Theorem 1 in Louizos’ original paper about the Y-X independence assumption, and the appendix for why proxy variables may introduce bias. We suggest either exploring how sensitive the method is for these deviations from the underlying model using realistic simulations, or showing why the method should work in additional graphical models that are in line with the presented analysis.

Q1: The lack of causal effect from X to Y in the causal model.

**Response:** Thanks for your question. We totally agree with you that there exists a relationship from X (observed confounders) to Y (outcome). Besides, we also regard X as the proxy variable of Z( unobserved hidden confounder) and learn the distribution of Z from X through a Variational AutoEncoder model. On the other hand, from the perspective of the generative model, the inference network as an encoder intends to learn the posterior representation $q(z|m,x,y)$ with the input sample $(m,x,y)$. Although Z is a hidden variable, it is also the carrier of information about X in this model. And the information of X can be sent to Y through Z. Hence, we don’t put an arrow from X to Y and remain only one arrow from Z to Y in the causal model shown in Fig. 1.

Q2: “Proxy variables may introduce bias.”

**Response:** We agree with you that proxy variables may introduce bias. With this in mind, we designed the last two terms in Eq. 6 to reduce the difference between the prior and the posterior of Z as much as possible and simultaneously reduce bias from proxy variables.

**2 The definition of the outcome (Y) should be better motivated and improved**

While we agree with the biological importance of the explored pathways in this paper, we think that the definition of Y should be improved. First, in theory, the proposed method can be run using the expression profile of any target gene g. By exploring all genes the authors can establish if a pathway of interest tends to be enriched among the genes with the strongest causal effects. This will avoid some of the arbitrary decisions made by the current definition of Y that relies on regression analysis of the most correlated gene set within the pathway of interest. We see the current definition as problematic for two reasons: (1) what if the explored pathway P is not highly correlated and can be clustered into two or more groups of co-expressed gene sets? as the presented methods CEBP is introduced as a general method for analyzing new data and not just the presented pathways in the paper, we feel that the assumption that the pathway of interest P must necessarily be driven by a single large group of co-expressed genes should be avoided whenever possible; (2) the identification of the main gene set within the pathway P relies on "Then we choose half the number of the features with higher gcor which can be considered as significant ones", why is this definition correct? This definition means that a set of “significant” genes will appear in any randomly generated data matrix, even if no true signal is present.

Q1: Questions on the current definition of Y and What if the explored pathway P is not highly correlated and can be clustered into two or more groups of co-expressed gene sets?.

**Response:** Thanks for your question. Our aim is to identify key mutations for important cancer-related biological processes. Followed by the definition of driver mutations which are “confer growth advantage” to cells and promote abnormal proliferation and migration, we only focus on the related biological processes but not other pathways. On the other hand, the so-called cancer-related biological process, it suggests that at least some of these important genes are activated to bring the overall activity level of the process up or down. The genes in a cancer-related biological process or pathway usually work together and are highly correlated under an active state. Even though they may be clustered into two or more groups, the largest cluster can carry the most information of biological processes, which is analogous to the first principal component of principal component analysis. What’s more, core genes are selected to facilitate the downstream regression analysis on each sample. If genes are not high-correlated, it will enlarge the residual error of regression and leads to converge difficultly. Of course, as you said, this is a general method, but we want to say that this should be in the context of biological processes or pathways, not other scenarios. Finally, we want to emphasize that the main contribution of CEBP is that it eliminates both observed and unobserved confounders and is expected to estimate the pure effect of a mutation on a cancer-related biological process. As a comparison, most existing computational methods for the identification of driver mutation are correlated-based and biased by confounders.

Q2: "Then we choose half the number of the features with higher gcor which can be considered as significant ones", why is this definition correct?

**Response:** Thank you for this question. Following with Q1, we plot the heatmap of the correlation matrix of each biological process with correlation p-value =10^-3^ as a cutoff, as shown in Fig. 1. It shows that more than half the number of genes of DNA replication and EMT are significantly co-expressed in the tumor samples of BRCA, LUAD and LIHC, respectively. X-axis and Y-axis represent gene’s index which is ranked by the overall co-expression intensity with other genes. Of course, the hyper-parameter “half the number of genes” can be adjusted when applying it to other tasks.

Figure 1. The correlation of genes in DNA replication and EMT of cancers.

**3 The proposed method does not estimate the variance of the causal effects**

A major limitation of the proposed method is that it only provides the point estimates but not their variance. Thus, it is difficult to estimate the false discovery rate of a set of selected results. We propose considering bootstrap for estimating the variance of these estimates.

**Response:** Thanks for your really good suggestion. We have repeated the experiment for 10 times and present the full experimental results including their mean and variance value in the revised version. The variance also shows that mutations with a higher ATE have a relatively small variance.

**4 The analysis should cover additional scenarios, datasets, and algorithms**

We think that the presented analysis is limited, and fails to establish the benefits of the proposed method. We think that the authors should apply the method to additional pathways or use all genes as we suggest above. Then the authors can show which pathways are likely to be affected by mutations and which do not. Moreover, applying pathway enrichment analysis downstream (e.g., when Y represents single genes) can be used to illustrate how the method can find novel biological insights without the need to select a specific Y in advance. Another major limitation of the proposed analysis is the lack of references to existing methods. While not directly relying on causal inference of observational data, multiple network biology methods have been proposed in the past for identifying causal connections between gene mutations and gene expression data. Early publications include the ResponseNet algorithm (Yeger-Lotem et al. 2009) and TieDIE (Paull et al. 2013), and additional algorithms have been proposed since. Comparing the output of these established methods with what causal inference methods can provide is of great interest for the community, and we believe will be a natural question for most readers. We also think that these methods should be explained in the introduction, which will help readers in understanding how the problem of identifying key mutations has been addressed in the past.

**Response:** Thank you for this suggestion. Our aim is to identify key mutations of oncogenesis through the causal connection of genomics change and phenotype change reflected by transcriptomic data. As you mentioned, many biological processes are indeed changed if we conduct differential expression analysis and pathway enrichment analysis when comparing cancer samples with normal ones. But we're not sure if these changed biological processes are actually cancer-related. Thus, it is not our focus in this current manuscript. The reason why we only choose DNA replication and EMT process is that they are consistent with the definition of the effect of driver mutations on cancer. There is no denying that you have made a good suggestion, it just needs more systematic analysis and a clear definition of meaningful biological processes. We are committed to working on the relevant studies.

We really appreciate your suggestion about adding some early methods as references, such as ResponseNet algorithm (Yeger-Lotem et al. 2009) and TieDIE (Paull et al. 2013), to make the introduction more complete. We have revised this part. Here, we also want to clarify that although these methods attempt to define driver mutation from a causal view or establish a causal link between genome and transcriptome, to the best of our knowledge, CEBP is the first method to identify key mutations based on the confounder-free causality mechanism.
